# Supplementary material for: Probing the symmetry of the potential of localized surface plasmon resonances with phase-shaped electron beams
Source: Nat Commun. 2017 Apr 12;8:14999. doi: 10.1038/ncomms14999 (PMC5394338; doi:10.1038/ncomms14999)
Supplement: Supplementary Information — Supplementary Figures, Supplementary Notes and Supplementary References [file ncomms14999-s1.pdf]

Supplementary Note 1. MORE THEORY (I): DERIVATION OF EQUATION 2

As shown in[1], the Green function has a convenient form in the case of a nanoparticle with dielectric function $\epsilon(\omega)$ in vacuum, for which a modal decomposition exists in terms of the eigenpotential $\Phi_m(\mathbf{r})$, and spectral function $g_m(\omega)$:

$$G(\mathbf{r}, \mathbf{r}', \omega) = \sum_m g_m(\omega)\phi_m(\mathbf{r})\phi_m^*(\mathbf{r}') \tag{1}$$

Injecting this equation in expression 15 of[2] we can obtain a rather compact form for the transition probability

$$\begin{aligned} \mathit{\Gamma}(\omega) \propto \sum_f \sum_m \Im(-g_m(\omega)) \left| \int \mathit{\Psi}_f(\mathbf{r})\phi_m(\mathbf{r})\, \mathit{\Psi}_i^*(\mathbf{r})\; d^3\mathbf{r} \right|^2 \cdot \\ \cdot\, \delta(\epsilon_f - \epsilon_i + \omega) \end{aligned} \tag{2}$$

In order to proceed further, we need to specify a form for the incident electron beam. Following the regular procedure[2,3], we simplify the calculations by supposing that 1: the beam's transverse momentum is negligible compared to the parallel one; 2: that it propagates along the optical axis z with a plane wave like behaviour; and 3: that the scattering follows the non-recoil approximation. Then the beam's wave function becomes

$$\mathit{\Psi}(\mathbf{r}) \propto \mathit{\Psi}_\perp(\mathbf{r}_\perp) \exp ik_z z \tag{3}$$

Thanks to this we can sum on all final state wavevectors $k_{f,z}$ which, due to the non-recoil approximation, results in the disappearance of the Dirac δ term in equation 2 (that expressed energy conservation), and we finally retrieve the equation 2. In this expression it becomes apparent how the transition between the two free electron states is mediated by the complex eigenpotential of the plasmonic resonance.

Supplementary Note 2. MORE THEORY (II): BEAM SHIFT

The description of the shifted probe is easily obtained, by operating the substitutions $(x) \to (x - x_0)$ and $(y) \to (y - y_0)$ in equation 3 which becomes:

$$\mathit{\Psi}_{i,\perp}(x, y) \propto (x - x_0) \exp\left(-\frac{(x-x_0)^2+(y-y_0)^2}{w^2}\right) \tag{4}$$

Consequently, equation 5 then becomes:

$$\Gamma(\omega) \propto \sum_m \Im(-g(\omega)) \cdot \\ \cdot \left| \int (x - x_0) \exp\left(- \frac{(x-x_0)^2 + (y-y_0)^2}{w^2} \right) \tilde{\phi}_m(x, y, q_z) \, dx dy \right|^2 \tag{5}$$

Supplementary Note 3. MEASUREMENT OF THE PHASE SHIFT INDUCED BY THE NEEDLE

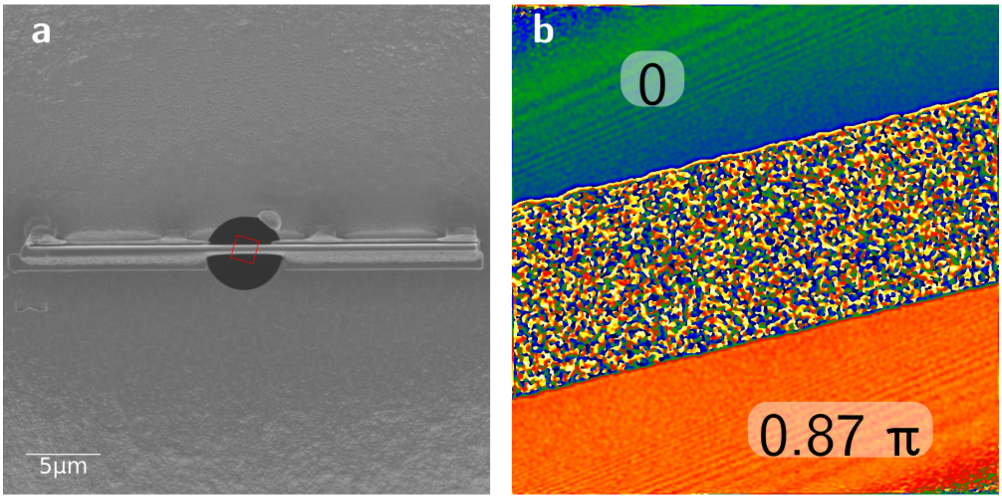

Supplementary Figure 1. Measurement of the phase shift induced by the needle. The red square in the scanning electron micrograph of the modified aperture (a) marks the region over which the phase difference was measured through electron holography . (b) shows the phase retrieved from the hologram. The fringes that can be observed at the top and bottom of the image are well known instrumental artefacts due to the near field diffraction of the electron wave at the edges of the Möllensted biprism, and do not correspond to a local phase modulation.

Supplementary Note 4. EFFECT OF PHASE IMPERFECTION ON BEAM SHAPE

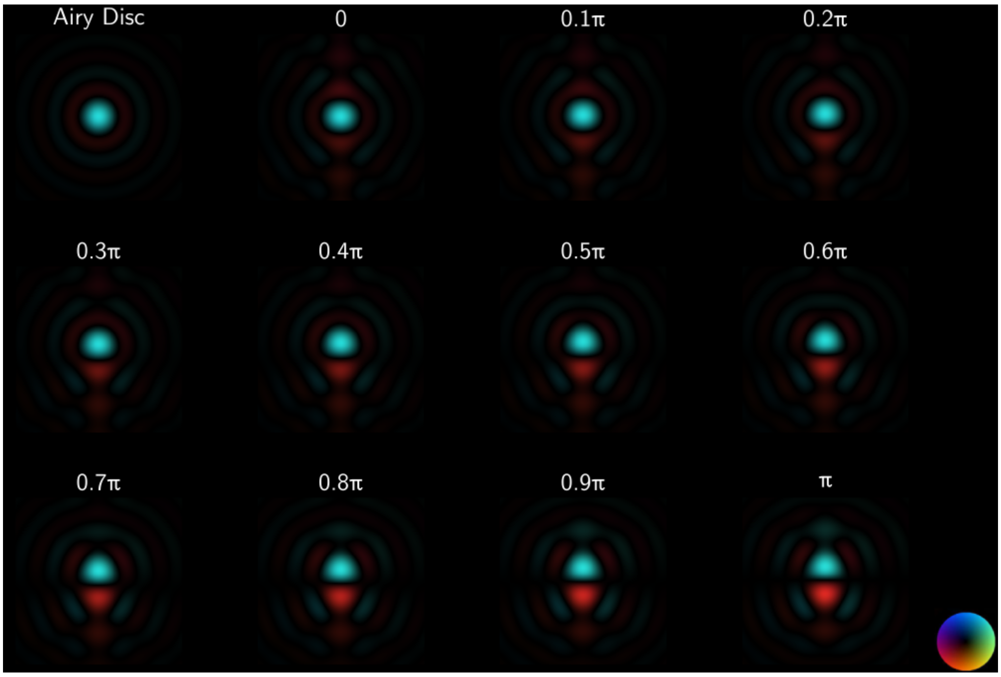

Supplementary Figure 2. Simulated electron wave functions for different values of phase shift. The first panel shows a simulated conventional electron probe, while all the successive ones show simulations of the beam's wave function for a modified aperture with a finite thickness needle. The simulations are ordered for increasing value of phase shift between the two sides of the aperture. The colour intensity represents wave function amplitude while the hue represents its phase, in accordance with the colourwheel.

Since the wave function of the probe directed on the sample is the Fourier transform of the wave function in the condenser aperture plane, any alteration in phase affects also the intensity distribution. This can be clearly seen in the figure above. The first panel shows an Airy disc, which is the Fourier transform of a circle and the wave function of a conventional beam. The successive panels, from the second to the last, contain simulations of the beam wave function for a modified aperture such as the one used in this work (including a needle of finite thickness), for different values of phase difference. It can be seen that the wave function is perfectly symmetric for a phase difference of 0, exactly antisymmetric for a phase difference of exactly π, and possesses varying degrees of asymmetry for intermediate values. In all panels the colour intensity encodes the wave function amplitude, and the

colour hue represents the phase, according to the colourwheel in the bottom right corner.

Supplementary Note 5. COUPLED NANOPARTICLES: SIMULATION OF A NANOROD–DIMER

The coupling of identical nanoparticles at short distance, result in the hybridisation of the plasmon modes of the individual particles.

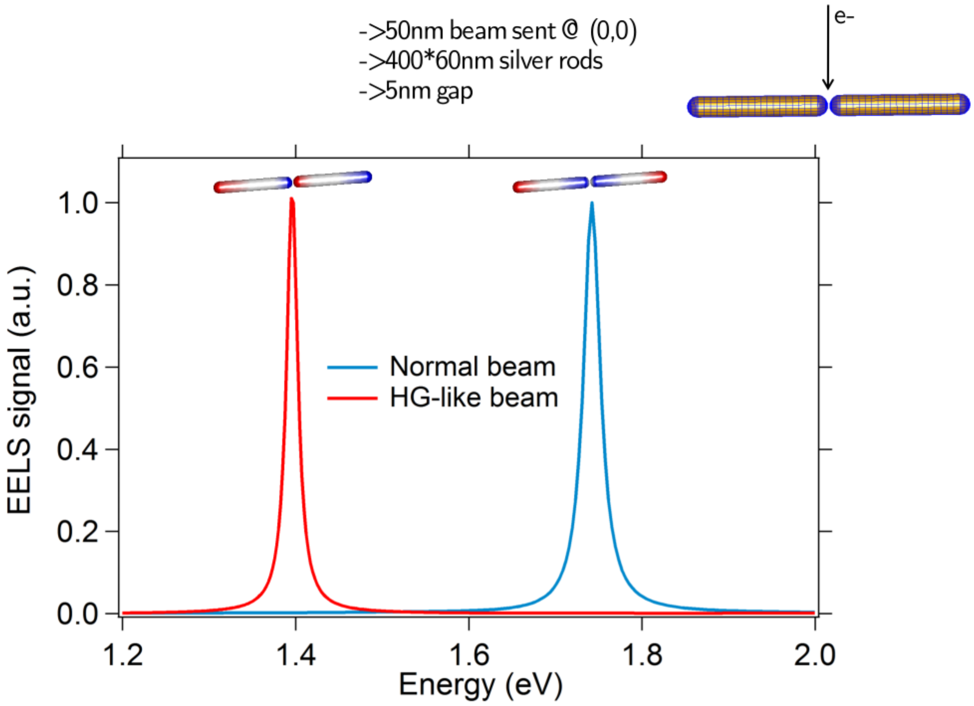

Supplementary Figure 3. Simulated spectra for normal and 2–lobed beams impinging on a nanoparticle dimer. I can be seen how the conventional and modified beam are predicted to probe the antisymmetric and symmetric states respectively.

This phenomenon splits the original dipolar mode into a symmetric and antisymmetric mode, with an energy that is shifted upwards or downwards respectively. Depending on the distance between the particles (and thus the coupling), the energy shift can be below the energy resolution of conventional EELS. By employing phase shaped beams however, the two modes can be directly separated, as shown in these simulations.

SUPPLEMENTARY REFERENCES

[1] G. Boudarham and M. Kociak, Physical Review B **85**, 245447 (2012).

[2] F. J. García de Abajo, Reviews of Modern Physics **82**, 209 (2010).

[3] A. Asenjo-Garcia and F. J. García de Abajo, Physical Review Letters **113**, 066102 (2014).
